# Inhibition of RNA Recruitment and Replication of an RNA Virus by Acridine Derivatives with Known Anti-Prion Activities

**DOI:** 10.1371/journal.pone.0007376

**Published:** 2009-10-13

**Authors:** Zsuzsanna Sasvari, Stéphane Bach, Marc Blondel, Peter D. Nagy

**Affiliations:** 1 Department of Plant Pathology, University of Kentucky, Lexington, Kentucky, United States of America; 2 USR3151-CNRS “Protein Phosphorylation & Human Disease”, Station Biologique, B.P. 74, 29682 Roscoff cedex, Bretagne, France; 3 INSERM U613, Brest, France; 4 Univ Brest, Faculté de Médecine et des Sciences de la Santé, UMR-S613, Brest, France; 5 Etablissement Français du Sang (EFS) Bretagne, Brest, France; 6 CHU Brest, Hop Morvan, Laboratoire de Génétique Moléculaire, Brest, France; Yale University, United States of America

## Abstract

**Background:**

Small molecule inhibitors of RNA virus replication are potent antiviral drugs and useful to dissect selected steps in the replication process. To identify antiviral compounds against Tomato bushy stunt virus (TBSV), a model positive stranded RNA virus, we tested acridine derivatives, such as chlorpromazine (CPZ) and quinacrine (QC), which are active against prion-based diseases.

**Methodology/Principal Findings:**

Here, we report that CPZ and QC compounds inhibited TBSV RNA accumulation in plants and in protoplasts. In vitro assays revealed that the inhibitory effects of these compounds were manifested at different steps of TBSV replication. QC was shown to have an effect on multiple steps, including: (i) inhibition of the selective binding of the p33 replication protein to the viral RNA template, which is required for recruitment of viral RNA for replication; (ii) reduction of minus-strand synthesis by the tombusvirus replicase; and (iii) inhibition of translation of the uncapped TBSV genomic RNA. In contrast, CPZ was shown to inhibit the in vitro assembly of the TBSV replicase, likely due to binding of CPZ to intracellular membranes, which are important for RNA virus replication.

**Conclusion/Significance:**

Since we found that CPZ was also an effective inhibitor of other plant viruses, including Tobacco mosaic virus and Turnip crinkle virus, it seems likely that CPZ has a broad range of antiviral activity. Thus, these inhibitors constitute effective tools to study similarities in replication strategies of various RNA viruses.

## Introduction

Viruses, including plant viruses, cause many diseases that lead to significant economic losses. Therefore, it is important to develop antiviral strategies to efficiently combat viral infections. One intriguing approach is the use of small molecule inhibitors of viral infections as demonstrated by the use of ribavirin against hepatitis C virus (HCV), respiratory syncytial virus and treatment of Lassa fever as well as amantadine and rimantadine M2 channel inhibitors and zanamivir and oseltamivir neuraminidase inhibitors against influenza virus [1], [2], [3]. Additional 40 antiviral drugs have been approved for treatment of human immunodeficiency virus and herpesviruses [2]. To find additional inhibitors of viral infections, researchers screened small molecule libraries that identified a few more potent antiviral chemicals [4], [5], [6], [7], [8], [9].

In spite of the above progress with small molecule inhibitors of human and animal viruses, identification of small molecule inhibitors to combat plant virus infections is much less advanced. Therefore, we have chosen a simple model virus, *Tomato bushy stunt virus* (TBSV), to test small molecule inhibitors. TBSV, a small (+)RNA virus of plants, has emerged as a highly suitable model virus for studying viral RNA replication and recombination [10]. The genomic (+)RNA of TBSV codes for p33 and p92^pol^ replication proteins [10]. p33 is an essential replication co-factor performing recruitment of the viral RNA template into replication [11], [12], [13], and in the assembly of the viral replicase [14]. The p92^pol^ is the RNA-dependent RNA polymerase (RdRp) [15], [16], [17]. Both replication proteins are integral part of the tombusvirus replicase complex, which also contains host proteins [18], [19]. Several of these host factors have been identified via proteomics approaches [19], [20], [21]. The list includes the heat shock protein 70 chaperones (Ssa1/2p in yeast), glyceraldehyde-3-phosphate dehydrogenase (GAPDH, encoded by *TDH2* and *TDH3* in yeast), pyruvate decarboxylase (Pdc1p), Cdc34p ubiquitin conjugating enzyme and eukaryotic translation elongation factor 1A (eEF1A) [19], [20], [21]. These host factors provide various functions, such as facilitating the assembly of the viral replicase complex and promoting asymmetrical viral RNA synthesis [22], [23], [24], [25].

One of the major advantages of tombusviruses is the development of yeast as a model host for virus - host interactions, allowing the utilization of powerful genomics and proteomics tools developed for yeast [16], [24]. Systematic genome-wide screens covering 95% of yeast genes have led to the identification of over 100 host factors affecting TBSV RNA replication [20], [21], [26], [27]. One of the surprising findings from these genome-wide screens is the observation that TBSV co-opt some host factors that also affect prion propagation, such as protein chaperones and ribosomal proteins [20], [21], [26], [27].

Based on the above observation, we have tested in this paper if inhibitors of prion propagation active against both yeast and mammalian prions [28] could also reduce TBSV replication. We found that two acridine derivatives, namely chlorpromazine (CPZ) and quinacrine (QC), inhibited TBSV RNA accumulation significantly in single plant cells. Besides their anti-prion effect, QC and CPZ have been used in humans for many years, respectively, as antimalarial and antipsychotic drugs [29], [30], [31]. Detailed studies on the mechanism of inhibition revealed that inhibition by CPZ and QC was due to effects of these compounds on the following steps: (i) inhibition of the assembly of the viral replicase complex (for CPZ), suppression of production of viral replication proteins during translation (for QC); (ii) inhibition of RNA–viral protein interaction during RNA recruitment for replication (for QC); and reducing minus-strand synthesis (for QC). Altogether, these acridine derivatives could be useful antiviral compounds against selected plant viruses.

## Results

### Acridine Derivatives Chlorpromazine and Quinacrine Inhibit TBSV RNA Accumulation in *N. Benthamiana* Protoplasts

**Figure 1:**
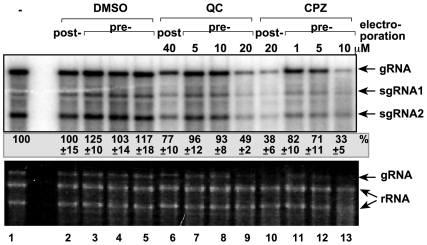
Inhibition of TBSV gRNA accumulation in *N. benthamiana* protoplasts by treatment with CPZ or QC. Northern blot analysis with a 3′ end specific probe was used to detect the accumulation levels of TBSV gRNA and subgenomic (sg)RNAs. The *N. benthamiana* protoplasts were treated with the shown concentrations of CPZ and QC either before or after electroporation. The samples were harvested 40 hours after electroporation. The ethidium-bromide stained gel at the bottom shows the ribosomal (r)RNA levels as loading controls. As a control, DMSO, the solvent for CPZ and QC, concentrations were the following: 0.04%, lanes 2,6; 0.005%, lanes 3,7, 12; 0.01%, lanes 4,8,13; and 0.02%, lanes 5 and 9. Note that the gRNA can reach rRNA levels in *N. benthamiana* protoplasts. The survival of the plant cells (after electroporation and treatment) was checked by measuring rRNA levels in total RNA extracts.

We have tested two acridine derivatives chlorpromazine (CPZ) and quinacrine (QC), which are known anti-prion molecules active against both yeast [28] and mammalian prions [29], [30], [31] for their effects on TBSV replication in plant protoplasts. The TBSV genomic (g)RNA was introduced via electroporation into *Nicotiana benthamiana* protoplasts, whereas CPZ and QC were added to the culture media either prior to electroporation or after electroporation (Fig. 1). We have applied the chemicals in various concentrations to test for their optimal antiviral effects as well as for possible cytotoxic effects on the *N. benthamiana* cells (not shown). We found that both CPZ and QC inhibited TBSV accumulation by ∼70 and 50%, respectively, when applied prior to electroporation (Fig. 1, lanes 9 and 13). CPZ was also effective in reducing TBSV RNA accumulation by 60% in protoplasts when applied after electroporation, albeit in higher concentration than used for pre-electroporation (Fig. 1, lanes 10 versus 13). QC was not able to reduce TBSV accumulation as efficiently when applied after electroporation (Fig. 1, lane 6). Altogether, these in vivo experiments have established that both CPZ and QC have anti-TBSV effects in single plant cells.

### CPZ and QC Inhibit TBSV RNA Replication In Vitro

**Figure 2:**
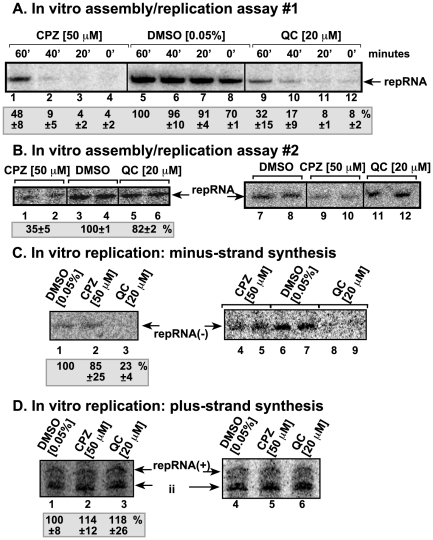
Inhibition of in vitro replication of TBSV repRNA in a cell-free extract by CPZ and QC. (A) The first in vitro replication assay contained a yeast cell-free extract, purified recombinant p33 and p92^pol^, a mixture of radiolabeled and unlabeled nucleotides and was programmed with TBSV DI-72 (+)repRNA. CPZ, QC and DMSO were added to the assay at 0, 20, 40 or 60 min after the start of the assay, which lasted for 3 hours. The newly made repRNA products were analyzed on denaturing PAGE. Note that the repRNA goes through a full replication cycle in this replication assay, producing mostly (+)-stranded progeny RNAs. (B) The second in vitro replication assay was similar to that described in panel A, except that the membrane fraction of the yeast cell-free extract was treated with CPZ, QC or DMSO first, followed by a washing step to remove the unbound compounds. This was followed by the addition of the soluble fraction of the yeast cell-free extract, purified recombinant p33 and p92^pol^, a mixture of radiolabeled and unlabeled nucleotides and was programmed with TBSV DI-72 (+)repRNA. DMSO concentrations were the following: 0.05%, lanes 1, 2, 3, 7, 9, 10 and 0.02% in lanes 4, 5, 6, 8, 11, and 12. The experiments were done in duplicates and the right panel shows reproducibility. The data show the averages and standard deviations including both sets. (C) The third in vitro replication assay contained a partially purified tombusvirus replicase preparation, a mixture of radiolabeled and unlabeled nucleotides and was programmed with TBSV DI-72 (+)repRNA. Note that the repRNA can only produce the complementary (minus-stranded RNA) product in this assay. (D) The fourth in vitro replication assay was similar to that described in panel C, except (−)-stranded repRNA was used to generate (+)-stranded RNA products. Note that the solubilized/purified tombusvirus replicase also generates internally initiated (marked as “ii”) products on (−)RNA template.

To define the mode of action for these acridine derivatives leading to inhibition of TBSV RNA accumulation, we took advantage of efficient in vitro replication assays developed for tombusviruses. First, we used an in vitro replicase assembly assay that includes purified recombinant TBSV p33 and p92^pol^ replication proteins and a yeast cell-free extract [23]. This cell-free system can be programmed with plus-stranded (+)TBSV replicon (rep)RNA, which leads to the assembly of the TBSV replicase on the membranes. The in vitro assembled replicase is capable of supporting complete cycle of TBSV replication leading to the production of (+)repRNA progeny [23], [32]. Testing the effect of CPZ and QC revealed 90–95% inhibition of TBSV replication when the chemicals were applied at an early time point of the assay (Fig. 2A, lanes 2–4 and 10–12). In the in vitro replicase assembly assay, the first 40 min consist of recruitment of the repRNA, the viral replication proteins and host proteins, such as Hsp70 to the membrane [23], [32], followed by the assembly of the membrane-bound replicase complex, whereas viral RNA synthesis occurs after ∼20–40 min of incubation until ∼3 hours. These experiments revealed that both CPZ and QC inhibited TBSV repRNA replication when added at the beginning of incubation (0 time point) (Fig. 2A, lanes 4 and 12), while they were not as effective at the 60-min time point (lanes 1 and 9). This suggests that CPZ and QC likely inhibit the early steps in virus replication, possibly including the recruitment of the viral RNA or the assembly of the functional replicase complex in the membrane, while they could only partially inhibit viral RNA synthesis in vitro.

### CPZ Inhibits the Formation of the TBSV Replicase Complex In Vitro

The viral replication proteins have to be associated with membranes for the functional assembly of the tombusvirus replicase [12], [32]. CPZ is known to bind to phospholipids and modify the features of membranes [33]. Therefore, we have tested if treatment of the membranes by CPZ could affect the subsequent assembly of the TBSV replicase. First, we pretreated the membrane fraction of the yeast cell extract for 15 min with either CPZ, QC or DMSO as a control, and then we washed the membrane fraction with buffer A to remove the chemicals not bound to membrane lipids. Second, to the pre-treated membrane fraction, we added the TBSV (+)repRNA and the purified recombinant p33 and p92^pol^ proteins in combination with the soluble fraction of the yeast extract. Under these conditions, the TBSV replicase can be successfully assembled in vitro and accordingly, replication of TBSV (+)repRNA was detected in the in vitro replication assay in the presence of the control DMSO (Fig. 2B, lanes 3–4 and 7–8). Interestingly, pre-treatment of the membrane fraction with CPZ led to 3-fold decrease in the TBSV replicase activity in vitro (Fig. 2B, lanes 1–2 and 9–10), whereas pre-treatment with QC had less than 20% inhibitory effect (lanes 5–6 and 11–12). These results demonstrated that pre-treatment of the membrane with CPZ inhibits the subsequent assembly of the TBSV replicase complex.

### QC Inhibits Minus-Strand Synthesis by the Tombusvirus Replicase

To test if CPZ and QC can inhibit minus- or plus-strand synthesis by the tombusvirus replicase, we also used an in vitro replicase assay based on solubilized and partially purified tombusvirus replicase from infected plants [34]. This solubilized tombusvirus replicase can use exogenous (added) template for complementary RNA synthesis only [34], [35]. When programmed with (+)repRNA template, the activity of the solubilized and partially purified tombusvirus replicase was reduced to 23% by QC treatment (Fig. 2C, lanes 3, 8, 9), whereas CPZ had only minor effect (lanes 2, 4, 5) when compared with the DMSO treated sample (lanes 1, 6, 7). On the other hand, neither compounds inhibited the activity of the solubilized and partially purified tombusvirus replicase programmed with a (−)repRNA template (Fig. 2D, lanes 2–3 and 5–6). Based on these data, we suggest that QC can selectively inhibit minus-strand synthesis, whereas the plus-strand synthesis is unaffected. On the other hand, CPZ does not seem to inhibit (−) or (+)-strand synthesis significantly by the tombusvirus replicase in vitro.

### QC Inhibits the Binding of p33 Replication Protein to the Viral (+)RNA

**Figure 3:**
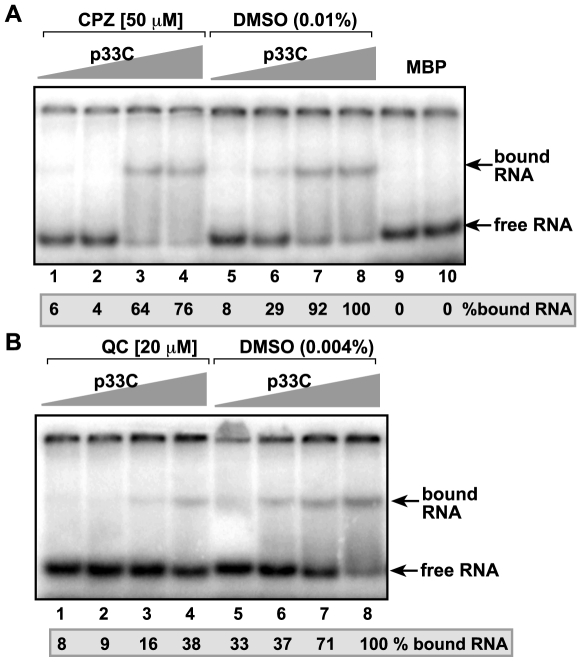
Inhibition of in vitro binding of p33 replication co-factor to TBSV (+)RNA by QC and CPZ. (A) The in vitro band-shift assay included the radiolabeled RII(+)-SL sequence, which is present within the p92 ORF in the TBSV gRNA, and 1.0, 0.4, 0.2 or 0.1 µg of purified recombinant p33C as well as CPZ (50 µM) or DMSO (0.01% final concentration). p33C is a soluble, N-terminally truncated version of p33 (contains the RNA-binding domain as well) fused to MBP. Purified recombinant MBP was used as a negative control. The binding was analyzed in 5% nondenaturing PAGE. The bound RNA in the DMSO control (lane 8) was chosen as 100%. Quantitation showed that the free RNA negatively correlated with the bound RNA. (B) Similar in vitro band-shift assay using QC (20 µM final concentration) as an inhibitor.

Previous works defined that binding of p33 replication protein to an internal recognition element, termed p33RE, which consist of a C•C mismatch within an internal stem-loop structure RII(+)-SL, is absolutely essential for TBSV replication in plants, plant protoplasts, in yeast cells and in the authentic in vitro TBSV replication system [11], [13], [32]. The binding of p33 to RII(+)-SL is required for template selection, the recruitment of the viral RNA for replication and for assembly of the active replicase complex. To test if binding of p33 to RII(+)-SL is affected by CPZ and QC, we performed in vitro gel-shift experiments with purified components. This assay is suitable to obtain information on binding of p33 to the viral RNA, which is relevant for in vivo TBSV RNA recruitment activity of p33 [13]. We found that QC strongly inhibited p33-RII(+)-SL interaction in vitro (Fig. 3B, lanes 1–4 versus 5–8), whereas the effect of CPZ was less pronounced at high p33 concentrations (Fig. 3A, lanes 3–4). Altogether, these in vitro binding experiments suggest that QC can strongly interfere with binding of p33 to the TBSV (+)RNA, thus inhibiting the recruitment of (+)repRNA into replication, whereas the effect of CPZ on the interaction might only be effective at lower p33 concentrations (e.g., in the beginning of infection).

### QC Inhibits Translation of Uncapped and Nonpolyadenylated TBSV RNA

**Figure 4:**
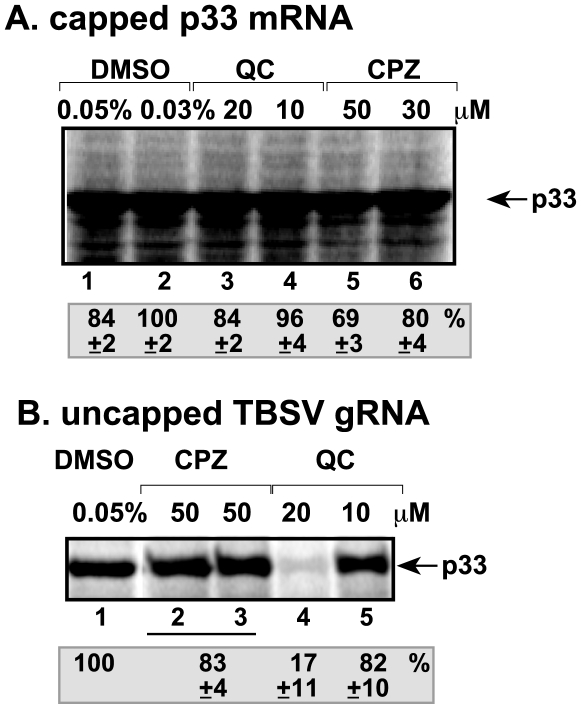
Inhibition of in vitro translation of TBSV RNA by QC. (A) The wheat germ translation assay was programmed with artificial capped p33 mRNA carrying a poly(A) tail in the presence of CPZ, QC and DMSO as control. The radiolabeled p33 product was analyzed on SDS-PAGE. Each experiment was repeated three times. DMSO sample (0.03%) was chosen as 100%. (B) Similar in vitro translation assay was programmed with the uncapped wt TBSV gRNA, which does not carry a poly(A) tail.

Another potential target for CPZ and QC is inhibition of translation of viral (+)RNA to produce p33 and p92^pol^ that would lead to reduced amount of replicase complex and low level of replication. To test this possibility, we used the in vitro wheat germ translation assay that can be programmed with added RNA templates. When capped and polyadenylated mRNA for p33 (similar to that expressed in yeast from plasmid to support TBSV replication) [16] was used, then we did not observe any major inhibitory effect by CPZ and QC (Fig. 4A, lanes 3–6). In contrast, in vitro translation of uncapped and nonpolyadenylated TBSV RNA, which is the common form of TBSV RNA replicating in *N. benthamiana* protoplasts (Fig. 1A), was inhibited by QC at 20 µM concentration resulting in ∼15% p33 when compared with the DMSO-treated control (Fig. 4B, lanes 4 versus 1), whereas CPZ did not have significant inhibitory effect (lanes 2–3). Thus, QC seems to selectively inhibit the translation of uncapped and nonpolyadenylated TBSV gRNA.

### CPZ and QC Inhibit TBSV RNA Accumulation in *N. Benthamiana* Plants

**Figure 5:**
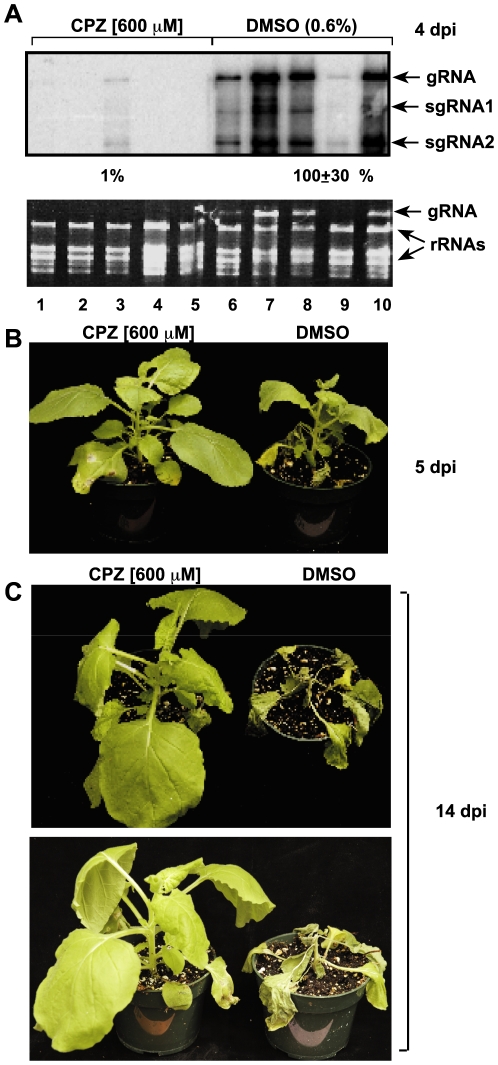
Inhibition of TBSV gRNA accumulation in *N. benthamiana* plants treated with CPZ. (A) Leaves were first infiltrated with CPZ (600 µM), followed by inoculation of the same leaves with TBSV virion preparation. Samples for viral RNA analysis were taken from the infiltrated leaves at 4 dpi. Northern blotting (top panel) shows the level of TBSV gRNA and sgRNAs accumulation in individual samples using a 3′ end specific probe. The bottom panel shows the ethidium bromide stained gel indicating the levels of rRNA and TBSV gRNA. Each experiment was repeated three times. DMSO sample was chosen as 100%. (B–C) The delay in symptom development due to TBSV infections in the CPZ treated plant (shown on the left) 4 and 14 dpi indicates the potent antiviral activity of CPZ. Comparable DMSO treatment of plant leaves prior to inoculation with TBSV did not protect the plants from infection.

Based on the above in vitro experiments, CPZ and QC might inhibit TBSV replication directly, encouraging us to further test the antiviral activities of these compounds in plant leaves as well. Our preliminary experiments have indicated that both compounds were needed at higher concentrations for treatment of plant leaves (not shown) than for the protoplasts experiments (Fig. 1). Infiltration of leaves with 600 µM CPZ solution 30 min prior to inoculation of the same leaves with TBSV virion preparation resulted in 99% inhibition of TBSV RNA accumulation at 4 dpi (Fig. 5A). Interestingly, the CPZ treatment also inhibited the virus symptom formation (Fig. 5B–C) and the rapid death of the plants caused by the systemic invasion by TBSV RNA (Fig. 5C). The CPZ treatment had no visible effect on the young leaves, while the older leaves became wavy (not shown) when CPZ was applied at 600 µM concentration.

**Figure 6:**
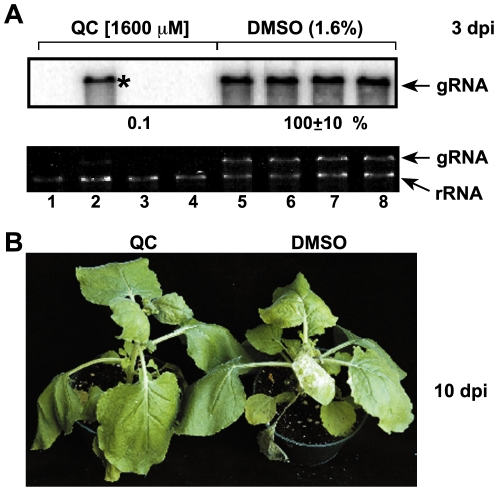
Inhibition of TBSV gRNA accumulation in *N. benthamiana* plants treated with QC. (A) Infiltration of leaves with QC (1600 µM), inoculation with TBSV and analysis of RNA samples were done as described under Fig. 5. Note that QC treatment resulted in delay of TBSV symptom formation when compared with DMSO-treated control plants. Note the 40% accumulation of TBSV RNA in one sample (marked with a star), which is likely due to areas in the leaf not (or partly) soaked by the compound during infiltration. Each experiment was repeated three times. DMSO sample was chosen as 100%. (B) The delay in symptom development due to TBSV infections in the QC treated plant (shown on the left) 10 dpi indicates an antiviral activity for QC when compared with the DMSO treatment of plant, which shows more severe symptoms (coloring and stunting).

Treatment of *N. benthamiana* leaves via infiltration of 1600 µM QC 30 min prior to inoculation with TBSV virion preparation also resulted in inhibition of TBSV RNA accumulation in the infiltrated leaves (by 99%, Fig. 6A). The formation of virus-induced symptoms was inhibited in QC-treated plants (Fig. 6B). However, the protection of the whole plants from TBSV infection was incomplete and QC-treated plants also developed the serious symptoms caused by TBSV infection with a delay of two-to-four days, followed by the death of the plants (not shown). Thus, the protection caused by QC treatment against TBSV infections is incomplete and temporary under the conditions used.

### CPZ Inhibits the Accumulation of Tobacco Mosaic Virus and Turnip Crinkle Virus in *N. Benthamiana* Protoplasts

**Figure 7:**
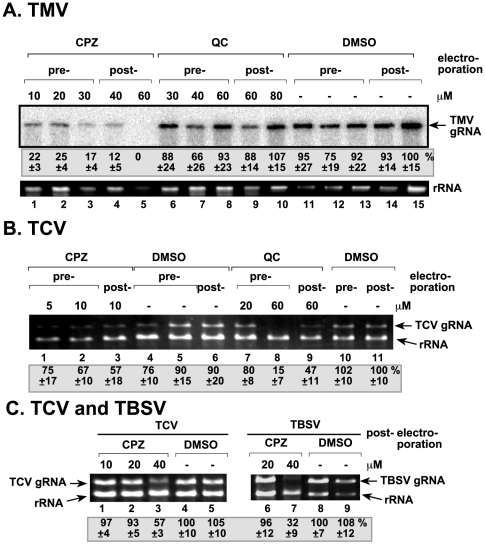
Inhibition of the distantly related TMV RNA and the closely related TCV RNA accumulation in *N. benthamiana* protoplasts by treatment with CPZ or QC. (A) Northern blot analysis with a 3′ end specific probe was used to detect the accumulation levels of TMV gRNA. *N. benthamiana* protoplasts were treated with the shown concentrations of CPZ and QC either before or after electroporation. See further details in the legend to Fig. 1. Note that the % of TMV RNA accumulation was normalized based on rRNA levels in the same samples to correct for sample-to-sample variation. DMSO concentrations were the following: 0.03%, lanes 3, 6, 11; 0.04%, lanes 4, 7, 12; 0.06%, lanes 5, 8, 9, 13, 14; and 0.08% in lanes 10 and 15. (B) Ethidium-bromide stained agarose gel electrophoretic analysis of the accumulation levels of TCV gRNA. *N. benthamiana* protoplasts were treated with the shown concentrations of CPZ and QC either before or after electroporation. See further details in the legend to Fig. 1. DMSO concentrations were the following: 0.005%, lanes 1, 4; 0.01%, lanes 2, 3, 5, 6; 0.02%, lanes 7, 10; and 0.06% in lanes 8, 9 and 11. (C) Comparison of the inhibitory effect of CPZ on TCV versus TBSV RNA accumulation. Ethidium-bromide stained agarose gel electrophoretic analysis of the accumulation levels of TCV and TBSV gRNAs from the same *N. benthamiana* protoplasts preparations. The protoplasts were treated with the shown concentrations of CPZ 30 min after electroporation. See further details in the legend to Fig. 1. DMSO concentrations were the following: 0.02%, lanes 2, 4, 6, 8; 0.04%, lanes 3, 5, 7 and 9.

To test if the effects of CPZ and QC are specific against TBSV, we also tested if these compounds can affect the replication of the related *Turnip crinkle virus* (TCV, *Tombusviridae*, flavivirus-supergroup) and the unrelated *Tobacco mosaic virus* (belonging to the alphavirus-supergroup of viruses) in *N. benthamiana* protoplasts. These experiments were conducted in a similar way to those shown for TBSV in Fig. 1. Interestingly, CPZ inhibited the accumulation of TMV RNA by ∼80% (Fig. 7A, lanes 3–4) and that of TCV by up to 43% (Fig. 7B–C). On the contrary, QC had only a small effect on TMV RNA accumulation (Fig. 7A, lanes 6–10), while QC treatment reduced TCV RNA accumulation by 90% when applied prior to electroporation in *N. benthamiana* protoplasts (Fig. 7B, lane 8). Direct comparison of the effect of CPZ and QC treatment on TCV and TBSV RNA accumulation in *N. benthamiana* protoplasts revealed that TBSV replication was more sensitive to CPZ treatment than TCV replication (Fig. 7C, compare lanes 3 and 7). Thus, while CPZ seems to have a broader antiviral effect, QC was inhibiting specifically TBSV and TCV RNA accumulation.

## Discussion

Small molecule inhibitors of virus replication are drug candidates to interfere with virus infections or cure viral diseases. This area is vastly understudied for plant viruses, though inhibitors against plant viruses might also be useful against animal viruses due to common replication mechanisms shared by many RNA viruses. In addition to the possible use of these compounds as antiviral agents, they could also be useful to understand various steps of virus replication and/or virus-host interactions.

The two acridine derivatives studied in this work for their anti-TBSV effects are CPZ and QC with known anti-prion [28], [36], [37], [38], [39], [40], anti-Alzheimer's beta-amyloid fibril formation [41], anti-malaria [42], anti*-Bacillus anthracis* toxin [43] and anti-cancer activities [44], [45]. Additional documented effects of CPZ include inhibition of the formation of clathrin-coated pits and the relocation of clathrin and clathrin-associated adaptor complex AP2 from the cell surface, which is critical for clathrin-mediated endocytosis used by severe acute respiratory syndrome coronavirus (SARS-CoV), dengue virus 2, Crimean-Congo hemorrhagic fever virus, vesicular stomatitis virus, HIV, mouse hepatitis virus type 2 during cell entry [46], [47], [48], [49], [50], [51], [52].

We have found that CPZ and QC affected TBSV replication. Although both compounds were the most effective when applied before or at the beginning of virus infection, detailed biochemical analysis of the mechanism of action by CPZ and QC revealed that they acted via different mechanisms.

**Figure 8:**
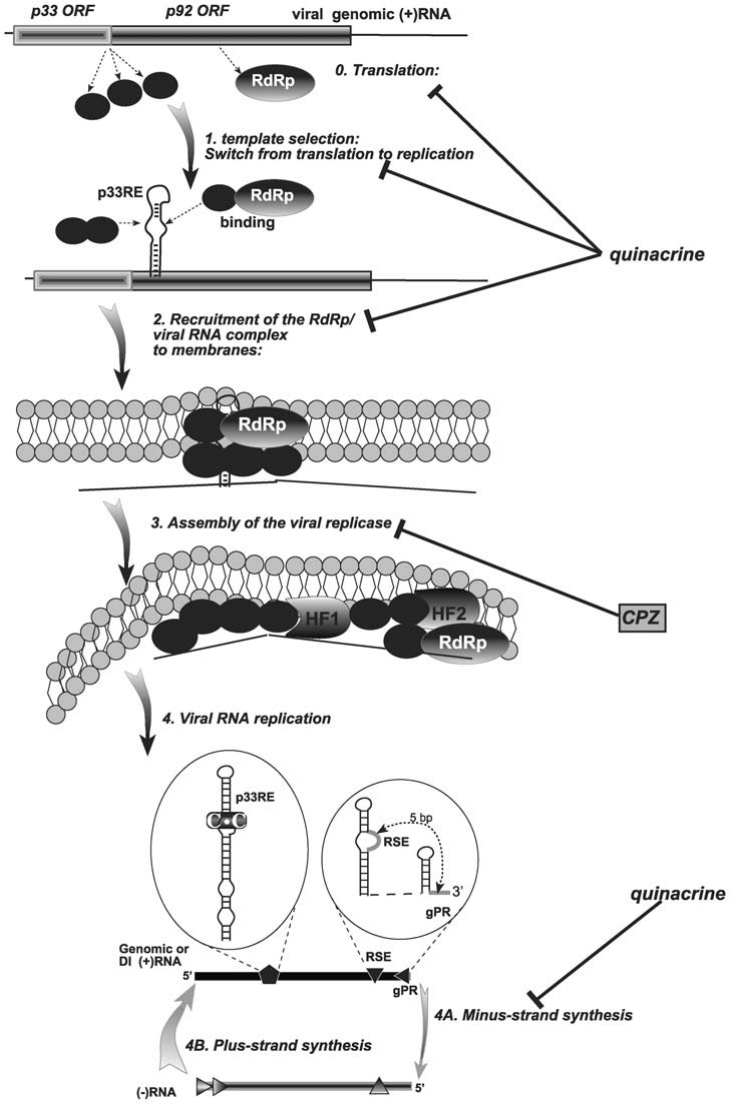
The proposed inhibitory effects of CPZ and QC treatments on various steps of TBSV RNA replication. Five early steps of TBSV replication [24], [70] are shown schematically, including translation of the viral RNA, template selection, viral RNA/protein recruitment into replication, assembly of the viral replicase complex and viral RNA synthesis. While the inhibitory effect of QC has been indicated in several steps, CPZ has been shown to affect mostly one step. The viral cis-acting RNA structures, such as p33RE (p33 recognition element), RSE (replication silencer element) and gPR (genomic promoter), which are likely bound by QC, are magnified.

In vitro RNA - protein binding experiments have shown that QC inhibited the binding of p33 replication protein to the internal replication element [p33RE consisting of the stem-loop RII(+)-SL] required for recruitment of the TBSV RNA into replication [11], [13]. The reduced level of viral RNA recruitment by p33 caused by QC is expected to lead to low level of replicase assembly, which requires the viral RNA component [14], [32], [53]. QC also inhibited minus-strand synthesis, but not (+)-strand synthesis, suggesting that QC selectively interacts with the viral (+)RNA template. Indeed, the RNA binding feature of QC has been demonstrated before including GAAA tetraloop [54], various base-paired RNAs [55], A-form and H(L)-form of poly(rC)-poly(rG) [56], and single-stranded poly(A) [57]. In addition to the above steps, QC also inhibited the translation of TBSV genomic RNA, which is uncapped and nonpolyadenylated. This inhibition was specific, since QC did not inhibit the translation of the capped/polyadenylated p33 mRNA in the wheat germ assay (Fig. 4). Altogether, although QC can inhibit various steps during the infectious cycle of TBSV (Fig. 8), it is possible that most or all of these inhibitory effects are due to the likely ability of QC to bind to viral RNA and inhibit protein - RNA interactions essential for TBSV replication.

The inhibitory effect of QC on TBSV replication is unlikely due to a putative general cytotoxic effect, since TMV RNA replication in protoplasts was not inhibited by QC under similar conditions used in the TBSV studies (Fig. 7A). TMV RNA is capped, so its translation might not be inhibited by QC. Similar to TBSV, the replication of the related TCV was inhibited by QC efficiently, especially when applied prior to electroporation (Fig. 7B).

The in vitro experiments have revealed that CPZ inhibits TBSV replication using a different mechanism from that of QC. We found that CPZ reduced drastically the in vitro activity of the TBSV replicase at the early time points, but CPZ had only weak inhibitory effect on binding of p33 to (+)RNA. In addition, CPZ did not inhibit the translation of TBSV gRNA. Also, CPZ did not inhibit (−) or (+)strand synthesis in an assay with the solubilized/purified tombusvirus replicase (Fig. 2C–D). In vitro replication of the TBSV repRNA was reduced only moderately by CPZ when used at a late (60 min) time point (Fig. 2A), arguing that once most of the replicase complexes have been assembled (between 0–60 min), then it becomes less sensitive to the presence of CPZ. Based on these data, it is likely that CPZ inhibits the assembly of the TBSV replicase, which occurs after translation and template selection/recruitment of the viral (+)RNA (Fig. 8). Albeit the assembly process is not yet completely understood for tombusviruses or other (+)-strand RNA viruses, it is likely that multiple viral protein - host protein and viral RNA - protein interactions as well as protein - subcellular membrane interactions have to take place for successful replicase assembly [24], [58]. Although CPZ could affect many of the above interactions, we obtained some evidence that interactions of the above factors with the subcellular membranes might be affected by the CPZ treatment (Fig. 2B). This is based on pre-treatment of the membrane fraction of the yeast cell-free extract with CPZ, followed by thorough washing of the membrane to remove unbound chemicals prior to the addition of the viral RNA template, the viral replication proteins, and the cytosolic (soluble) fraction of the cell-free extract. The pre-treatment of the membrane fraction with CPZ has led to reduced level of TBSV replication in vitro (Fig. 2B). Since CPZ binds rapidly to phospholipid bilayers and disturbs the molecular ordering of phospholipids [33] and CPZ is also known to affect the bending of the membrane lipids by favoring outward bending of membranes, we propose that CPZ could inhibit the assembly of the replicase complex and thus, TBSV replication via altering the features of intracellular membranes. Indeed, CPZ, when inserted into lipid bilayers, has been shown to induce spontaneously positive curvatures, and inhibit the formation of surfaces with negative curvature [59]. However, replication and the assembly of the replicase of some RNA viruses needs bending of the membranes in inward direction to assemble viral-induced spherules, where replication takes place [60], [61]. So, the opposing bending effect of CPZ and viral replication proteins during the replicase assembly might lead to reduced efficiency of viral replicase assembly. However, we cannot rule out that CPZ could bind to some membrane proteins present in the membrane fraction of the cell extract, which could also lead to a decrease in viral replicase assembly.

Therefore, the respective effects of QC and CPZ against plant viruses parallel their putative mode of action on prion propagation. Indeed, antiprion activity of both QC and CPZ may be related to their ability to down regulate the protein folding activity of the ribosome (Ribosome-borne Protein Folding Activity or RPFA) [62], [63]. Interestingly, QC is able to directly interact with Domain V of the large rRNA (which bears the RPFA that may be involved in prion propagation) whereas the effect of CPZ would be more indirect and involves its ability to interact with and disturb cell membranes thus activating a transduction pathway ultimately leading to down regulation of the synthesis of rRNA and also of other ribosome constituents (MB, unpublished). The parallelism with the situation observed here (QC having a role through a direct interaction with RNA, CPZ having a more indirect role involving its capacity to interfere with cell membrane) is particularly noticeable. The analysis of the effect on plant viruses of other antiprion compounds also able to interfere with RPFA and RNA is therefore of interest and will be the subject of future studies.

The effect of CPZ on cellular membranes can also explain why the treatment with CPZ is so effective in whole plant experiments as well as against the related TCV and the distantly related TMV, which also requires subcellular membrane for its replication [64]. Moreover, CPZ has been shown to inhibit the assembly of enveloped viruses [65], [66], which also require membrane bending. In contrast, the effect of QC seems to be narrower and includes plant viruses without cap and poly(A) ends, such as TBSV and TCV, members of *Tombusviridae*, a large family of plant viruses.

## Materials and Methods

### Chemicals

Chlorpromazine (CPZ) and quinacrine (QC) were purchased from Sigma. Both compounds were dissolved in DMSO and stored at −20°C as a stock at concentration of 100 mM. Further dilutions were made with water just before use.

### Preparation and Electroporation of *N. Benthamiana* Protoplasts

For protoplast isolation, *N. benthamiana* callus culture was treated with 1 g cellulysin and 0.2 g macerase (Calbiochem) as described [17]. Freshly prepared protoplasts were electroporated with 2 µg T7 RNA polymerase transcribed TBSV, TMV or TCV genomic RNAs as described [17], [25]. CPZ and QC dissolved in DMSO were added at various concentrations as shown in the figures to protoplasts 30 min before or 1 hour after electroporation of the viral RNAs. In the control samples, DMSO was added to the protoplasts at similar concentrations to that present in CPZ or QC preparations. Incubation of protoplasts was done in the dark at room temperature.

### Analysis of TBSV, TMV and TCV RNA Accumulation in *N. Benthamiana* Protoplasts

Total RNA was extracted 40 hrs after electroporation, followed by agarose gel analysis and Northern blotting using radiolabeled TBSV probe as described [17]. The template for the TMV RNA probe was generated by PCR using the full-length cDNA for TMV and primers #2890 (5′-TCTGGTTTGGTTTGGACCTC-3′) and #2889 (5′-GTAATACGACTCACTATAGGGATTCGAACCC-3′). The normalization of samples was done based on quantified ribosomal RNA levels in total RNA samples visualized by ethidium-bromide staining of gels.

### In Vitro TBSV Replication Assay Based on Yeast Cell-Free Extract

Cell-free yeast extract was prepared and replication assay for TBSV DI-72 RNA was performed as described [23]. For the treatment of the membrane fraction of the yeast cell-free extract, first, we separated the membrane fraction from the soluble fraction by centrifuging 20 µl of yeast extract at 21,000 g for 10 min at 4°C. Second, the membrane fraction was treated for 15 min at 25°C with CPZ or QC at the final concentration of 50 µM and 20 µM, respectively. To remove the unbound excess compounds from the membrane fraction, we washed the membrane fraction twice with 100 µl buffer A (30 mM HEPES-KOH, pH 7.4, 100 mM potassium acetate, and 2 mM magnesium acetate). After the wash step, we performed the TBSV replication assay for 3 hours at 25°C using the above membrane fraction in combination with the untreated soluble fraction according to [23].

### In Vitro Tombusvirus Replicase Assay for Minus- and Plus-Strand Synthesis in the Presence of CPZ and QC

For the preparation of the tombusvirus replicase, we used *N. benthamiana* plants infected with *Cucumber necrosis virus*, a close relative of TBSV, which can replicate TBSV RNA templates as efficiently as the TBSV replicase [34]. After preparing the membrane fraction containing the active CNV replicase, we solubilized the replicase and partially purified it as described [34]. The obtained tombusvirus replicase is template dependent and it can use added TBSV-derived RNA templates for complementary RNA synthesis, including (+) or (−)RNA synthesis depending on the polarity of the added RNA template [34], [35], [67]. We performed the replicase assay for 3 hours at 25°C using (+) or (−)DI-72 RNA in the presence of 50 µM CPZ, 20 µM QC or DMSO (0.01% and 0.004%, respectively) as a control. The RNA products obtained were analyzed by denaturing 8 M urea PAGE as described [34], [35], [67].

### In Vitro Assay for Testing the Binding of p33 Replication Protein to the Viral RNA in the Presence of CPZ and QC

The sequence for RII(+)-SL RNA (containing the cis-acting p33RE that is involved in selective binding to p33) was amplified with PCR using primers #1300 ( 5′- GTAATACGACTCACTATAGAGGTTTGTGAGAAGGTTGG- 3′) and #1301 (5′-CTGGCTCGTGTGTAAGTACG-3′) and DI-72 cDNA clone as a template [68]. Radioactively labeled RII(+)-SL RNA was obtained using T7 polymerase transcription as described [13]. MBP tagged p33C [13] and MBP, as a control, were affinity purified from *E. coli* [69]. The recombinant proteins were used in 1, 0.2, 0.4, and 0.1 µg for incubation with 1 ng of radioactively labeled RII(+)-SL RNA [13] in a binding buffer [12 mM HEPES pH 7.9, 15 mM KCl, 0.2 µM DTT, 10% glycerol, 2 U of RNase inhibitor (Ambion)] [13], [69] in the presence or absence of 50 µM of CPZ, 20 µM of QC or DMSO (0.01% and 0.004%, respectively) at 25°C for 15 min. Samples were analyzed by 5% non-denaturing polyacrylamide gel electrophoresis at 4°C ran at 200 V.

### Wheat Germ Cell Free Extract-Based in Vitro Translation of TBSV RNA

Uncapped, nonpolyadenylated TBSV genomic RNA transcripts were obtained with the T7 transcription using linearized DNA template [68]. Capped and polyadenylated p33 mRNA was obtained with the T7 transcription using PCR template made with primers # 2144 (5′-GTAATACGACTCACTATAGGG AAGCTATACCAAGCATACAATC-3′) and #2145 (5′-TTTTTTTTT-TTTTTTTTTTTTTTTTTTTTTTTTTTTTTTTTTTTAAAACCTAAGAGTCAC-3′). In vitro translation of RNA transcripts was carried out in a wheat germ extract (Promega) according to manufacturer's recommendation. Briefly, 0.3 pmol of RNA transcripts, 3 µl wheat germ extract and 0.3 µl amino acid mix without methionine (provided by the manufacturer) were mixed in 50 mM potassium acetate with a final volume of 6 µl. In addition, 1 µl of CPZ (final concentration of 30 µM or 50 µM), QC (10 or 20 µM), and DMSO (0.05%) was also added. Reactions were conducted for 1 hour in the presence of [^35^S] methionine (1000 Ci/mmol) at room temperature. In vitro translation was stopped by denaturation at 100°C for 3 minutes. Products were analyzed using 10% SDS-PAGE and quantified by Typhoon 9400 and ImageQuant (GE Healthcare).

### RNA Analysis of TBSV Accumulation in *N. Benthamiana* Plants Treated with CPZ or QC

Two leaves of *N. benthamiana* plants (four leaf-stage) were infiltrated with 2 ml of a 600 µM solution of CPZ or of a 1600 µM solution of QC. Control plants were infiltrated with the same percentage of DMSO that was present in the CPZ and QC solutions. The infiltrated leaves were inoculated with a purified TBSV virion preparation 30 min after infiltration. Total RNA was extracted 4 days post inoculation (dpi) and 1% agarose gel electrophoresis was done to analyze viral and ribosomal RNAs. Northern blot analysis was performed with a TBSV specific probe as described above for the protoplast experiments.
